# Experiences of Rural-Dwelling Children Wearing Physical Activity Trackers: An Exploratory Study

**DOI:** 10.3390/children11091108

**Published:** 2024-09-11

**Authors:** Katy Bray, Mengyuan Hao, Veronica Lelo, Heather Katz, Kristen A. Pickett, Susan J. Andreae

**Affiliations:** 1 Kinesiology Department, University of Wisconsin-Madison, Madison, WI 53706, USA; 2 Program in Occupational Therapy, University of Wisconsin-Madison, Madison, WI 53706, USA

**Keywords:** rural, children, physical activity, objective measures, qualitative

## Abstract

Background/Objectives: Although there is a need for evidence-based physical activity programs in rural communities, evaluating such programs is often challenging due to access-related barriers and measurement tools that are not designed for rural contexts. This study aimed to explore and better understand the day-to-day experiences of rural-dwelling children using wrist-worn PA trackers as part of a study to develop a health promotion program. Methods: Ten caregivers and child dyads were enrolled (*n* = 20). The children wore accelerometers pre- and post-intervention. Semi-structured interviews were completed post-intervention and were audio recorded, transcribed, and summary reports were generated based on recurring themes. Results: The children had a mean age of 8.7 (SD = 1.4) years and the majority were male (80%). The caregivers were female, white, and had a mean age of 43.6 (SD = 8.5) years, with an annual income of ≥USD 40,000. Factors contributing to device wear times included low caregiver burden, device functioning as a watch, and device interactivity. The children reported that the devices were acceptable, but may have changed their physical activity behaviors, with children regularly checking their step count. The caregivers preferred devices that monitored the children’s activity levels without sharing location data. Conclusions: Identifying acceptable and feasible strategies to measure physical activity is vital to developing effective health promotion efforts. The lessons learned may help develop evaluation plans for implementing rural physical activity programming.

## 1. Introduction

Physical activity (PA) is a modifiable behavior that improves health and wellness [1] and reduces the risk of chronic conditions [2,3]. However, significant disparities remain in creating equitable access to opportunities for engaging in PA in rural communities [4]. Rural U.S. communities include approximately 20% of the population [5] and are often socially and geographically isolated [6], are more likely to live in obesogenic environments [7,8,9], and there is limited availability and access to health resources [8,10,11,12,13,14]. These barriers may contribute to lower levels of PA [15,16,17,18] and a higher prevalence of childhood obesity in rural communities compared to non-rural areas [19,20,21].

Despite ongoing efforts to improve access to health promotion programs addressing the unique barriers to health faced by rural residents, additional work is required to create long-term change. The need remains for initiatives that are culturally responsive, while considering the individual, social, and environmental contexts of rural communities [22,23]. The development of health promotion programs that support health-enhancing behaviors, such as PA, requires measurement methods that provide valid and reliable data to inform program effectiveness. However, evaluating health programs in rural settings is often challenging due to access-related barriers and measurement tools that are not designed for rural communities [24,25]. PA measurement in children can be even more challenging as they are more likely to engage in sporadic, spontaneous activities throughout the day [26]. And, depending on their age, children have more difficulty providing accurate reports of their PA behaviors [26].

Access to effective health promotion programs that support the development of PA habits early in life potentially has long-term implications, as physically active children are more likely to be physically active adults [27,28]. Improving access to and the availability of such programs requires reliable and valid measurement strategies to inform the development of the program, the evaluation of program efficacy and effectiveness, and ongoing quality improvement efforts. For PA behaviors, the measurement tool selected is an important decision that can impact the PA levels [29]. For example, self-report methods may be more cost effective and easier to administer, especially for rural and remote communities, but they are limited by factors like bias related to the instrument, social desirability, and recall. Diary-based or direct measurement methods are more demanding in terms of participant burden and can be more costly and labor intensive to administer [26]. Hip-worn accelerometers can be more demanding in terms of participant burden and be higher in cost and have lower wear times and device adherence [30,31]. With the increased availability and improvements in commercially available wrist-worn fitness trackers, using these devices in research may be more cost effective, user friendly, and place less burden on the participants [32]. In short, the measurement tools and data collection protocols should consider participant and community-specific barriers to help ensure usability and data accuracy. Identifying acceptable and feasible strategies to accurately measure PA, a complex and multidimensional behavior, is necessary to effectively evaluate and monitor health promotion initiatives for communities that have been historically underrepresented in health research.

The aim of this study was to explore and better understand the day-to-day experiences of rural-dwelling children wearing PA trackers, which were used as part of a study developing and testing a health promotion program. Thus, our team conducted semi-structured interviews with children that participated in the study by wearing commercially available wrist-worn PA monitors and their caregivers as part of a lifestyle change program for rural-dwelling families at risk of type 2 diabetes. The interviews were used to better understand the children’s daily experiences, focusing on the feasibility and acceptability of using a commercially available wrist-worn fitness tracker for program evaluation purposes.

## 2. Methods

### 2.1. Participant Recruitment and Data Collection

This report presents the results from a secondary analysis of data collected as part of a pilot study on a family-based intervention for mothers and their children at risk of type 2 diabetes, conducted in Wisconsin. All study activities were reviewed and approved by the University Institutional Review Board. The participants were rural-dwelling caregivers and child (7–11 years old) dyads with a family history of type 2 diabetes, who were willing to participate in physical activities and group videoconference sessions using Zoom Version 5. Participants were excluded if they did not have regular access to a stable internet connection or had an injury or illness that would prevent participation in PA. The participants were recruited by distributing study flyers in the community and in partnership with the Survey of the Health of Wisconsin, a representative cohort study of Wisconsin residents [33,34]. Recruitment letters and emails were sent to individuals in communities defined as rural by the 2010 Census Urban and Rural Classification [35]. Enrollment occurred in Spring 2023.

Interested community members spoke to study staff on the telephone, were provided with study information, and were screened for eligibility. After speaking with the caregivers, the staff provided a study overview and assessed the child’s level of interest in participating in the study. Eligible and interested dyads were scheduled for baseline study visits. Data collection occurred at the baseline and after the intervention and lasted from 45 to 60 min. The data were entered into REDCap platform for management [36,37]. The adults received USD 40 at each data collection visit and the children kept their PA tracker after the study ended.

Before the baseline visit, participants were mailed the consent and assent forms and data collection materials. The visits began by obtaining signed consent from the adults and verbal assent from the children. Next, the adults completed assessments administered by a trained staff member. Then, the children were fitted with a wrist-worn fitness tracker that they were asked to wear for seven days. The caregivers were asked to complete a daily log and document the wear times and the reasons why the devices were removed. The participants then completed a 12-week lifestyle change program, which consisted of eight online group sessions that focused on family health and wellness, with topics including physical activity basics and benefits, reducing sedentary behaviors, healthy eating, and sleep health [38]. After completing the program, the participants repeated all the data collection activities. The follow-up visits also included a semi-structured interview with the participants to obtain program feedback. An interviewer guide was used to maintain consistency in the interviews. The questions focused on program content, readability, appropriateness, attractiveness, enjoyment, useability, and helpfulness. Specific questions regarding the PA trackers were included that explored how the participants interacted with the devices, what they liked or did not like about the devices, and any additional barriers and facilitators encountered. This report presents the findings from these interviews. The caregivers and children were interviewed separately. The semi-structured interviews were audio recorded and transcribed verbatim. The transcriptions were checked for completeness and accuracy by comparing the transcript to the audio recordings and de-identified in preparation for the analysis. The qualitative data were organized using NVivo [39].

### 2.2. Outcomes and Measures

The children wore an accelerometer, Garmin Vivofit Jr. 3, for seven days, for at least 12 h a day, on their non-dominant wrist. The devices were waterproof and had a battery that lasted up to two years and did not need to be recharged. Participants were asked to wear the trackers during all waking hours. If the device was removed during the day, the caregivers were asked to note in a log the times and the reason for removal. The device data were verified using the dates and times recorded in the log.

The caregivers reported the PA levels for their children using the 8-item Patient-Reported Outcomes Measurement Information System (PROMIS) Parent Proxy Pediatric Physical Activity Measure [40]. This measure is designed for children and youths aged 5–17 years old and provides a measure of the lived experience of PA over a 7-day reporting period, with higher scores reflecting higher levels of PA behaviors. Global health was measured using the 7-item PROMIS Pediatric Global Health Parent Proxy Scale, which is designed for children and youths aged 5–17 years old [41]. Family health was measured using the Family Health Scale. The measure includes 32 items that examine a family’s social and emotional health processes, healthy lifestyle, health resources, and external social support [42].

### 2.3. Analysis

Descriptive analysis of the baseline characteristics of the sample was conducted by creating frequency distributions of the categorical variables and the means and standard deviations of the continuous variables. Summary reports of the qualitative data, focusing on program feasibility and acceptability regarding the PA measurement protocols, were generated based on recurring themes found in the transcripts, guided by the six-step thematic analysis procedures described by Braun and Clarke [43]. The initial codebook, with codes and definitions, was developed by two coders working independently using the first transcript. These codes were discussed as a team, with differences reviewed and resolved through group consensus. The remaining interview transcripts were coded by two coders working independently using the codebook. After each transcript was coded, the coding teams met to review the application of the codes and to identify coding discrepancies. During the meetings, the discrepancies were discussed and a consensus was reached by the coders. After the meetings, the transcripts were recoded, if needed, to ensure consistent application of the codes. Then, the coded data were examined for patterns, similarities, and differences. Finally, the data were grouped into broad categories. To improve the rigor in the analysis process, multiple coders working independently were used to ensure the consistent application of the codes [44]. An audit trail was used to track the development of the codebook and categories [45].

## 3. Results

Ten caregivers and child dyads (*n* = 20) were enrolled, with nine dyads (*n* = 18) completing the program and participating in the follow-up data collection. The caregivers were on average 43.6 years old, female, white, had attended some level of college education, and had an annual income of USD 40,000 or greater (Table 1). There was an average of three children in each of the homes (range = 1–4). As seen in Table 1, the children were on average 8.7 years old, the majority were male (*n* = 8), and most were attending grades 3 or 4 (*n* = 6) in school. Most caregivers reported having excellent family health (*n* = 9). Moreover, 80% of caregivers reported average global health ratings for their child, with scores within one standard deviation of the reference population.

All the children wore their tracking device for seven complete days. The data showed that the mean minutes of activity over the seven days was 86.6 (SD = 19.6, range 64.3–124.3). The step count data showed a mean daily step count of 9846 (SD = 2567, range = 6745–15,398).

### 3.1. Device Acceptability and Feasibility

The semi-structured interview data focusing on the feasibility and acceptability of the trackers were summarized in terms of the following categories: (1) impact of the device on daily activities and device acceptability, (2) impact of the device on PA behavior, (3) caregiver experiences helping their children, and (4) summary of experiences of completing data collection visits remotely.

#### 3.1.1. Category 1: Devices Had Minimal Impact on Daily Activities and Were Acceptable to the Children

First, the feedback regarding the trackers was generally positive, with all nine children who completed the semi-structured interview noting that the devices had minimal impact on their daily activities. The participants noted that they liked that the devices functioned as watches, with seven participants specifically mentioning that they liked and used the watch and timer functions. Two children who already regularly wore a watch did not mind swapping their watch for the device during data collection. One child noted that the device had more functions compared to their current watch.

All the participants had complete wear and corresponding log data. The devices were waterproof and had a battery that lasted 1–2 years, so the children could leave the device on for the entire data collection period. The most frequently cited reasons for removal included sleeping, taking baths, and swimming. Most opted to remove the devices at night, with two children wearing them overnight. Reasons why the children opted not wear them during the night were that the devices were uncomfortable at night, or the device sensor light woke them up during the night. Eight children reported that they would be willing to wear the device for the entire study period (3 months) and planned to continue to wear the device after the study ended. One child was unsure whether they would be willing to wear a device regularly for an extended period, mainly because they preferred not to wear a watch every day.

Secondly, although most reported that the devices were acceptable in terms of size and comfortable to wear, two participants noted that they were not used to regularly wearing a watch and needed time to adjust. One child shared that when they first began wearing the device, they asked their caregiver if they could take it off, “…because my arm [felt] a little sore, so I had to take it off. And then, um, well, I took it off because, um, and I felt it was, it was like, I bounced and it was, or I, um, and dents from the watch, so, I took it off” (P08). Both participants reported that the issue was fixed after their caregiver helped them to loosen the device strap.

Finally, all the children noted that they liked the way the device looked, especially because it looked like and functioned as a watch. When asked if any of their friends noticed or asked about the devices, all the children reported that their friends did not notice the device. That the devices looked like and functioned as a watch may be an important factor to consider when selecting a device for use by children. Eight of the nine participants were unsure about wearing a device on their leg or waist (their caregivers wore an accelerometer on their waist, so the children were asked if they would be willing to wear a similar device). One child noted, “…um, no, I like it on my arm. It’s like my other spider[man] watch. Um, I showed them [friends] the movement and timer” (P03). Social acceptability may be a concern, with four participants specifically discussing that wearing a device on their leg or waist may prompt questions from their peers: “Not really, because it wouldn’t just like this [points to watch], they would ask what it is” (P09). Another asked if they could wear the device under their clothes, so the device was not visible, “…my mom put hers underneath her shirt. I [could] do that too?” (Interviewer then asks: “why under your shirt?”) Child responded, “You don’t see it” (P002).

#### 3.1.2. Category 2: Impact of the Device on PA Behavior

No participants had any previous experience of wearing an activity tracker before the study and shared that they were excited to wear the device. All the participants reported that they looked at their step count throughout the data collection period. Six reported that they regularly checked their step count during the day. Three participants specifically noted this information often motivated them to be more active, particularly while engaging in an activity. One child noted, “I liked that [the watch] checked my steps and it was kind of fun to try to get it higher and higher each day” (p008). In addition, two children who had family members who wore a PA tracker reported comparing their step counts with each other to see who had accrued the most steps at the end of each day, “We made a competition out of it that way. See who had more…it was fun” (P02).

However, for the other participants, the level to which their PA behavior changed is unclear. While they noted that they would look at their step counts, the tracking of such data did not necessarily prompt additional activity. For example, one child shared, “I would kind of look in like, every once in a while. Just to know how many steps I have”. When asked what happened if the steps were lower than they wanted or expected, the child responded, “Nothing” (P09). Another noted, “I liked [the watch] because I could see what time it is. And, like, I know how much that I walked in on one day”. When asked whether the number of steps recorded by the device ever made them do additional activities, the child noted, “I, um, there were days when I missed the goal, but I didn’t do more because I was very tired” (P11).

As there is mixed evidence in the literature regarding participant reactivity due to commercially available activity monitors [46,47,48,49], we examined the change in the mean daily step count over the seven days to see whether the novelty of wearing an activity tracker may have resulted in higher step counts at the beginning of the data collection period. However, we did not find any significant differences between the mean step counts or minutes of activity at the start of the 7 days compared to the end of the 7 days.

#### 3.1.3. Category 3: Caregiver Experiences Helping Their Children with the Devices

The caregivers shared that the time burden of completing the daily log and helping the children with the devices during the week was low and acceptable. All noted that it was not challenging to get their child to put on and wear the device the entire day, but the child required prompting on most days. The caregivers mentioned the importance of the device having a readable display and additional functions. They believed that these factors were important to improve adherence. Because of this level of engagement with the devices, all the caregivers discussed the potential to integrate the devices into intervention activities and not just as a data collection tool.

When discussing other potential types of monitoring devices, such as the ActiGraph wGT3X-BT device that the caregivers wore on their waist during the study, the caregivers had mixed reactions when asked about the possibility of asking their child to wear a similar device. They were concerned about their child’s willingness to wear the device consistently on their waist. They also expressed concerns about the device being lost or broken and noted that the children would require more assistance and monitoring during the data collection period. Three caregivers noted that the use of such devices may be possible if fewer data collection days were required and were completed during non-school days. And, while the caregivers were open to different types of devices, even ones that they would have to recharge regularly, five caregivers preferred devices that only monitored the step count, activity minutes, and heart rate, without being connected to the internet or sharing location data.

#### 3.1.4. Category 4: Summary of Experiences Completing Data Collection Visits Remotely

Because the enrolled families lived in rural communities, all the data collection activities for this study, including device orientation and fitting, were completed via video conferencing. The caregivers reported that they liked the flexibility offered for scheduling the visits, not having to travel to the data collection sites, and did not mind navigating the technical or logistical issues encountered. It is important to note that the study used the data collection visits as an opportunity to familiarize the participants with the video conferencing system in a one-on-one setting before beginning the group intervention. Thus, research staff were ready to assist the caregivers in accessing and navigating the application. The staff reached out by telephone, as needed, to help caregivers set up and log in to the application before the scheduled appointments and time was built into the visits to account for these activities. All but three caregivers used a smartphone or tablet to connect during the visits. One participant did not have a stable internet connection, so most of their visit was completed over the telephone, with only the device fitting and demonstration on how to use the device happening via video conference. Another caregiver required a follow-up call to review the instructions on how to use the daily log to document the wear times. The virtual study visits saved time and funds that would have been spent by study staff driving several hours to multiple rural towns and opened the program up to families in more remote regions. As noted by the participants, remotely conducting the visits provided greater flexibility for busy caregivers to schedule the visits during convenient times. All the visits were primarily completed on the weekends, early in the mornings, before school, or in the evenings, after school. However, additional time and resources were needed to mail the materials to the study participants and to ensure that staff were available to complete the virtual visits. Although these resources were built into the study budget and timeline, several packages were lost or delayed in the mail, requiring additional time and funds to resend the materials to the families. One child lost their device after removing it for swimming practice and another device broke during the week, delaying the intervention program start date by approximately two weeks. Finally, it is important to note that all the caregivers in our study already had some familiarity with the video conferencing application and had access to relatively stable internet connections.

## 4. Discussion

Our study explored the experiences of children wearing commercially available fitness trackers to collect objective PA data for a research study. The challenges of accurately measuring complex health behaviors, such as PA, in children are well-known [50,51]. Identifying acceptable and feasible strategies to measure PA accurately are vital to developing effective health promotion efforts in rural and other hardly reached communities. We spoke with caregivers and children with the goal of better understanding their preferences, concerns, the barriers, and facilitators to using wearable activity trackers to inform the protocols for future program evaluations. Our discussions centered around the following categories: (1) the impact of the device on daily activities and device acceptability, (2) the impact of device on PA behavior, (3) the caregiver’s experience helping their children, and (4) a summary of the experience of completing the data collection visits remotely.

First, the children reported positive experiences and that the devices did not interfere with their daily activities. All the children participating in the data collection had seven complete days of wear, with the most common reasons for removal being sleeping, taking baths, and swimming. For the children and caregivers in our study, the following factors were important for adherence: the devices functioned as watches, had a readable screen so the children could interact with the devices, and the appearance of the device was acceptable.

As our main goal was to use the devices to measure PA behavior for program evaluation, the semi-structured interviews explored the impact of the devices on the children’s behavior. There is mixed evidence in the literature regarding the impact of wearable activity trackers on changing PA behaviors [46,47,48,49]. In the studies that do show a change in PA outcomes, the reported change is often small. A recent meta-analysis of PA interventions that included the use of commercially available, wearable PA trackers as the primary intervention component or as a supplemental tool for monitoring behaviors as part of a broader lifestyle change program, found that there was an increase in the daily step count, moderate and vigorous PA, and energy expenditure [47]. However, the use of the devices only increased the overall daily step count by 627 steps [47]. Moreover, the programs that utilized PA trackers with the biggest impact on changing behavior were multifaceted and included additional behavioral change strategies [46,47,49].

Similarly, the children in our study discussed how the devices changed their behaviors, noting that they were excited to receive the trackers and that they regularly checked their step count during the week. However, the level to which their activity level changed due to the device is unclear. The average daily step count for our study was slightly lower compared to published normative data for boys (12,000 to 16,000 steps) and girls (10,000 to 13,000 steps) [52]. The caregiver-reported data showed average PA levels for their child, with all scores within one standard deviation of the reference population. While the children reported that they regularly checked their step count during the week, they also noted that the number did not necessarily prompt a change in their PA behavior. The degree to which using activity trackers to monitor PA in children may meaningfully change behaviors requires further investigation.

Finally, reports show that adherence and wear time of PA monitoring devices often decrease over time [46,53]. Although our sample is small, we explored factors with the children and caregivers that may contribute to adherence. These included having caregiver assistance and support, selecting devices that required lower caregiver burden by not requiring frequent recharging, the ability for the children to interact with the devices, and the fact that the devices looked like and functioned as watches. Likewise, studies that examine the use and adherence in terms of PA monitoring devices in children and youths report that wrist-worn devices may be more acceptable and increase wear times [48,54]. In addition, our data showed that selecting devices that minimized the burden on the caregivers and the ability for the children to interact with the devices may be essential. These are essential characteristics to consider during device selection and the development of measurement protocols.

Our study was strengthened by the engagement of rural-dwelling children and their caregivers to learn about their experiences to inform future study protocols that used PA monitoring devices. However, this study had several limitations. Our study sample included families living in multiple rural communities throughout Wisconsin. However, all the enrolled families were white, with caregivers who had taken part in higher education and had income levels compared to rural Wisconsin communities [35]. Self-reported measures of PA, global health, and family health were used and were limited by the risk of recall and social desirability bias. The study participants also had average PA levels and reported high family health. It is important that future studies include more diverse perspectives, especially of children who have lower PA levels.

Moreover, because of the small sample size and the nature of the qualitative data, our findings are not generalizable. Rather, they may represent the experiences of families living in similar contexts. Finally, our study only included families with access to stable internet connections, with higher levels of digital literacy. It is important to include the perspectives of families who are less comfortable with technology or those without access to stable internet connections, a common barrier for many rural residents [55,56]. Despite these limitations, our study provides insights into the impact of monitoring devices on PA behaviors and the factors related to the acceptability of using PA monitoring devices for research in rural communities.

## 5. Conclusions

Challenges remain in developing culturally relevant, acceptable, and feasible health promotion programs for rural communities. Further work is needed to identify best practices to objectively measure PA in children. This study explored the acceptability and feasibility of using wrist-worn activity trackers for PA measurement in rural-dwelling children. By engaging children and caregivers to gain their perspectives in the development and planning of the program evaluation process, we hoped to better understand the unique issues faced in specific rural contexts in order to inform the design and evaluation of health promotion programs. The lessons learned and approaches for objective PA measurement outlined in this report may help develop evaluation plans that balance study goals with the needs of rural communities, thereby increasing the access and availability of feasible and acceptable evidence-based opportunities to engage in health-enhancing behaviors like physical activity.

## Figures and Tables

**Table 1:** Baseline participant characteristics.

	Caregivers (*n* = 10)	Children (*n* = 10)
Age, mean ± SD	43.6 ± 8.5	8.7 ± 1.4
Sex, *n*		
Male participant	0	8
Female participant	10	2
Caregiver education, *n*	10 attended some level of college education or further studies	
Child grade level, *n*		
Grade 1 or 2		2
Grade 3 or 4		6
Grade 5 or 6		2
Household income, *n*	10 had income of ≥USD 40,000	
Children in each home, *n*	3 (range 1–4)	
Caregiver employment, *n*		
Working full time	7	
Working part time	2	
Not working	1	
Family Health Scale, *n* *		
Poor family health	0	
Moderate family health	1	
Excellent family health	9	
Global health of children, mean T-score ± SD **	47.6 ± 8.5	

* Family Health Scale [42]. ** The 7-item PROMIS Pediatric Global health, Parent Proxy Measure [41].

## Data Availability

The raw data supporting the conclusions of this article will be made available by the authors on request.

## References

[1] Marquez D.X., Aguiñaga S., Vásquez P.M., Conroy D.E., Erickson K.I., Hillman C., Stillman C.M., Ballard R.M., Sheppard B.B., Petruzzello S.J. (2020). A systematic review of physical activity and quality of life and well-being. Transl. Behav. Med..

[2] Aune D., Norat T., Leitzmann M., Tonstad S., Vatten L.J. (2015). Physical activity and the risk of type 2 diabetes: A systematic review and dose–response meta-analysis. Eur. J. Epidemiol..

[3] Chomistek A.K., Cook N.R., Rimm E.B., Ridker P.M., Buring J.E., Lee I. (2018). Physical Activity and Incident Cardiovascular Disease in Women: Is the Relation Modified by Level of Global Cardiovascular Risk?. J. Am. Heart Assoc..

[4] Kegler M.C., Gauthreaux N., Hermstad A., Arriola K.J., Mickens A., Ditzel K., Hernandez C., Haardörfer R. (2022). Inequities in Physical Activity Environments and Leisure-Time Physical Activity in Rural Communities. Prev. Chronic Dis..

[5] U.S. Department of Agriculture (2024). What Is Rural? Economic Research Service. https://www.ers.usda.gov/topics/rural-economy-population/rural-classifications/what-is-rural.

[6] Hussain B., Mirza M., Baines R., Burns L., Stevens S., Asthana S., Chatterjee A. (2023). Loneliness and social networks of older adults in rural communities: A narrative synthesis systematic review. Front. Public Health.

[7] Christiana R.W., Bouldin E.D., Battista R.A. (2021). Active living environments mediate rural and non-rural differences in physical activity, active transportation, and screen time among adolescents. Prev. Med. Rep..

[8] Wende M.E., Meyer M.R.U., Abildso C.G., Davis K., Kaczynski A.T. (2023). Urban-rural disparities in childhood obesogenic environments in the United States: Application of differing rural definitions. J. Rural Health.

[9] Hansen A.Y., Meyer M.R.U., Lenardson J.D., Hartley D. (2015). Built Environments and Active Living in Rural and Remote Areas: A Review of the Literature. Curr. Obes. Rep..

[10] Veitch J., Salmon J., Ball K., Crawford D., Timperio A. (2013). Do features of public open spaces vary between urban and rural areas?. Prev. Med..

[11] Wilcox S., Castro C., King A.C., Housemann R., Brownson R.C. (2000). Determinants of leisure time physical activity in rural compared with urban older and ethnically diverse women in the United States. J. Epidemiol. Community Health.

[12] Whitfield G.P., Carlson S.A., Ussery E.N., Watson K.B., Berrigan D., Fulton J.E. (2019). National-level environmental perceptions and walking among urban and rural residents: Informing surveillance of walkability. Prev. Med..

[13] Holmes M., Thompson K.W. (2019). Risk Factors and Potentially Preventable Deaths in Rural Communities.

[14] Meyer M.R.U., Moore J.B., Abildso C., Edwards M.B., Gamble A., Baskin M.L. (2016). Rural Active Living: A Call to Action. J. Public Health Manag. Pract..

[15] (2017). Rural Health Snapshot.

[16] Garcia M.C., Faul M., Massetti G., Thomas C.C., Hong Y., Bauer U.E., Iademarco M.F. (2017). Reducing Potentially Excess Deaths from the Five Leading Causes of Death in the Rural United States. MMWR Surveill. Summ..

[17] Matthews K.A., Croft J.B., Liu Y., Lu H., Kanny D., Wheaton A.G., Cunningham T.J., Khan L.K., Caraballo R.S., Holt J.B. (2017). Health-Related Behaviors by Urban-Rural County Classification—United States, 2013. MMWR Surveill. Summ..

[18] Anderson T.J., Saman D.M., Lipsky M.S., Lutfiyya M.N. (2015). A cross-sectional study on health differences between rural and non-rural U.S. counties using the County Health Rankings. BMC Health Serv. Res..

[19] Crouch E., Abshire D.A., Wirth M.D., Hung P., Benavidez G.A. (2023). Rural–Urban Differences in Overweight and Obesity, Physical Activity, and Food Security Among Children and Adolescents. Prev. Chronic Dis..

[20] Probst J.C., Barker J.C., Enders A., Gardiner P. (2016). Current State of Child Health in Rural America: How Context Shapes Children’s Health. J. Rural Health.

[21] Johnson J.A., Johnson A.M. (2015). Urban-Rural Differences in Childhood and Adolescent Obesity in the United States: A Systematic Review and Meta-Analysis. Child. Obes..

[22] Richman L., Pearson J., Beasley C., Stanifer J. (2019). Addressing health inequalities in diverse, rural communities: An unmet need. SSM-Popul. Health.

[23] Holmes C., Levy M. (2015). Rural Culture Competency in Health Care White Paper.

[24] Payton Scally C., Burnstein E., Gerken M., Immonen E. (2020). In Search of “Good” Rural Data.

[25] Pierce C., Scherra E. (2004). The Challenges of Data Collection in Rural Dwelling Samples. Online J. Rural Nurs. Health Care.

[26] Sylvia L.G., Bernstein E.E., Hubbard J.L., Keating L., Anderson E.J. (2013). Practical Guide to Measuring Physical Activity. J. Acad. Nutr. Diet..

[27] Dohle S., Wansink B. (2013). Fit in 50 years: Participation in high school sports best predicts one’s physical activity after Age 70. BMC Public Health.

[28] Telama R., Yang X., Leskinen E., Kankaanpää A., Hirvensalo M., Tammelin T., Viikari J.S.A., Raitakari O.T. (2014). Tracking of Physical Activity from Early Childhood through Youth into Adulthood. Med. Sci. Sports Exerc..

[29] Prince S.A., Adamo K.B., Hamel M.E., Hardt J., Gorber S.C., Tremblay M. (2008). A comparison of direct versus self-report measures for assessing physical activity in adults: A systematic review. Int. J. Behav. Nutr. Phys. Act..

[30] Gao Z., Liu W., McDonough D.J., Zeng N., Lee J.E. (2021). The Dilemma of Analyzing Physical Activity and Sedentary Behavior with Wrist Accelerometer Data: Challenges and Opportunities. J. Clin. Med..

[31] O’Brien W.J., Shultz S.P., Firestone R.T., George L., Breier B.H., Kruger R. (2017). Exploring the challenges in obtaining physical activity data from women using hip-worn accelerometers. Eur. J. Sport Sci..

[32] Henriksen A., Mikalsen M.H., Woldaregay A.Z., Muzny M., Hartvigsen G., Hopstock L.A., Grimsgaard S. (2018). Using Fitness Trackers and Smartwatches to Measure Physical Activity in Research: Analysis of Consumer Wrist-Worn Wearables. J. Med. Internet Res..

[33] Nieto F.J., Peppard P.E., Engelman C.D., McElroy J.A., Galvao L.W., Friedman E.M., Bersch A.J., Malecki K.C. (2010). The Survey of the Health of Wisconsin (SHOW), a novel infrastructure for population health research: Rationale and methods. BMC Public Health.

[34] Malecki K.M.C., Nikodemova M., Schultz A.A., LeCaire T.J., Bersch A.J., Cadmus-Bertram L., Engelman C.D., Hagen E., McCulley L., Palta M. (2022). The Survey of the Health of Wisconsin (SHOW) Program: An Infrastructure for Advancing Population Health. Front. Public Health.

[35] United States Census Bureau 2010 Census Urban and Rural Classification and Urban Area Criteria. U.S. Department of Commerce. https://www.census.gov/programs-surveys/geography/guidance/geo-areas/urban-rural/2010-urban-rural.html.

[36] Harris P.A., Taylor R., Minor B.L., Elliott V., Fernandez M., O’Neal L., McLeod L., Delacqua G., Delacqua F., Kirby J. (2019). The REDCap consortium: Building an international community of software platform partners. J. Biomed. Inform..

[37] Harris P.A., Taylor R., Thielke R., Payne J., Gonzalez N., Conde J.G. (2009). Research electronic data capture (REDCap)—A metadata-driven methodology and workflow process for providing translational research informatics support. J. Biomed. Inform..

[38] Andreae S.J., Lindberg A., Casey T., Pickett K.A. Developing and Pretesting a Family-Based Physical Activity Program for Rural Dwelling Mothers and Their Children at Risk for Diabetes. Health Serv Res Manag Epidemiol..

[39] QSR International (2018). Nvivo Qualitative Data Analysis Software, Version 12.

[40] Tucker C.A., Bevans K.B., Becker B.D., Teneralli R., Forrest C.B. (2020). Development of the PROMIS Pediatric Physical Activity Item Banks. Phys. Ther..

[41] Forrest C.B., Bevans K.B., Pratiwadi R., Moon J., Teneralli R.E., Minton J.M., Tucker C.A. (2013). Development of the PROMIS® pediatric global health (PGH-7) measure. Qual. Life Res..

[42] Crandall A., Weiss-Laxer N.S., Broadbent E., Holmes E.K., Magnusson B.M., Okano L., Berge J.M., Barnes M.D., Hanson C.L., Jones B.L. (2020). The Family Health Scale: Reliability and Validity of a Short- and Long-Form. Front. Public Health.

[43] Braun V., Clarke V. (2012). Thematic analysis. APA Handbook of Research Methods in Psychology.

[44] O’connor C., Joffe H. (2020). Intercoder Reliability in Qualitative Research: Debates and Practical Guidelines. Int. J. Qual. Methods.

[45] Nowell L.S., Norris J.M., White D.E., Moules N.J. (2017). Thematic Analysis: Striving to Meet the Trustworthiness Criteria. Int. J. Qual. Methods.

[46] Böhm B., Karwiese S.D., Böhm H., Oberhoffer R. (2019). Effects of Mobile Health Including Wearable Activity Trackers to Increase Physical Activity Outcomes Among Healthy Children and Adolescents: Systematic Review. JMIR mHealth uHealth.

[47] Brickwood K.-J., Watson G., O’Brien J., Williams A.D. (2019). Consumer-Based Wearable Activity Trackers Increase Physical Activity Participation: Systematic Review and Meta-Analysis. JMIR mHealth uHealth.

[48] Ridgers N.D., McNarry M.A., Mackintosh K.A. (2016). Feasibility and Effectiveness of Using Wearable Activity Trackers in Youth: A Systematic Review. JMIR mHealth uHealth.

[49] Casado-Robles C., Viciana J., Guijarro-Romero S., Mayorga-Vega D. (2022). Effects of Consumer-Wearable Activity Tracker-Based Programs on Objectively Measured Daily Physical Activity and Sedentary Behavior Among School-Aged Children: A Systematic Review and Meta-analysis. Sports Med.-Open.

[50] Howells K., Coppinger T. (2021). Children’s Perceived and Actual Physical Activity Levels within the Elementary School Setting. Int. J. Environ. Res. Public Health.

[51] Corder K., van Sluijs E.M., McMinn A.M., Ekelund U., Cassidy A., Griffin S.J. (2010). Perception Versus Reality: Awareness of Physical Activity Levels of British Children. Am. J. Prev. Med..

[52] Tudor-Locke C., Craig C.L., Beets M.W., Belton S., Cardon G.M., Duncan S., Hatano Y., Lubans D.R., Olds T.S., Raustorp A. (2011). How many steps/day are enough? for children and adolescents. Int. J. Behav. Nutr. Phys. Act..

[53] Fleur R.G.S., George S.M.S., Leite R., Kobayashi M., Agosto Y., Jake-Schoffman D.E. (2021). Use of Fitbit Devices in Physical Activity Intervention Studies Across the Life Course: Narrative Review. JMIR mHealth uHealth.

[54] Dobell A., Pringle A., Faghy M.A., Roscoe C.M.P. (2020). Fundamental Movement Skills and Accelerometer-Measured Physical Activity Levels during Early Childhood: A Systematic Review. Children.

[55] Perrin A., Duggan M. (2015). Americans’ Internet Access 2000–2015. Pew Research Center.

[56] Vogels E. (2021). Some Digital Divides Persist Between Rural, Urban and Suburban America. Pew Research Center.

